# Research Review: Why do prospective and retrospective measures of maltreatment differ? A narrative review

**DOI:** 10.1111/jcpp.14048

**Published:** 2024-08-16

**Authors:** Oonagh Coleman, Jessie R. Baldwin, Tim Dalgleish, Kelly Rose‐Clarke, Cathy Spatz Widom, Andrea Danese

**Affiliations:** ^1^ Social, Genetic and Developmental Psychiatry Centre, Institute of Psychiatry, Psychology and Neuroscience King's College London London UK; ^2^ Division of Psychology and Language Sciences, Department of Clinical, Educational and Health Psychology University College London London UK; ^3^ Medical Research Council Cognition and Brain Sciences Unit University of Cambridge Cambridge UK; ^4^ Cambridgeshire and Peterborough NHS Foundation Trust Fulbourn UK; ^5^ Institute for Global Health University College London London UK; ^6^ Psychology Department, John Jay College City University of New York New York NY USA; ^7^ Graduate Center City University of New York New York NY USA; ^8^ Department of Child and Adolescent Psychiatry, Institute of Psychiatry, Psychology and Neuroscience King's College London London UK; ^9^ National and Specialist CAMHS Clinic for Trauma, Anxiety, and Depression South London and Maudsley NHS Foundation Trust London UK

**Keywords:** Childhood maltreatment, childhood trauma, child abuse, neglect, memory, psychopathology

## Abstract

**Background:**

Childhood maltreatment contributes to a large mental health burden worldwide. Different measures of childhood maltreatment are not equivalent and may capture meaningful differences. In particular, prospective and retrospective measures of maltreatment identify different groups of individuals and are differentially associated with psychopathology. However, the reasons behind these discrepancies have not yet been comprehensively mapped.

**Methods:**

In this review, we draw on multi‐disciplinary research and present an integrated framework to explain maltreatment measurement disagreement.

**Results:**

We identified three interrelated domains. First, methodological issues related to measurement and data collection methods. Second, the role of memory in influencing retrospective reports of maltreatment. Finally, the motivations individuals may have to disclose, withhold, or fabricate information about maltreatment.

**Conclusions:**

A greater understanding of maltreatment measurement disagreement may point to new ways to conceptualise and assess maltreatment. Furthermore, it may help uncover mechanisms underlying maltreatment‐related psychopathology and targets for novel interventions.

Child maltreatment has been identified as an important risk factor for poor mental and physical health outcomes, including depression (Brown, Cohen, Johnson, & Smailes, 1999), behavioural problems (Widom, 1989), psychotic symptoms (Arseneault et al., 2011), and obesity (Danese & Tan, 2014). Identification of this at‐risk group therefore has important research and clinical implications, and the way in which child maltreatment is defined, measured, and identified has drawn considerable attention (Danese, 2020; Kendall‐Tackett & Becker‐Blease, 2004; Widom, Raphael, & DuMont, 2004). In particular, child maltreatment can be measured based on prospectively or retrospectively collected information. Prospective measures are collected while the child is growing up and typically rely on parental or official reports, which capture a third‐person perspective. In contrast, retrospective measures are often collected in adulthood and rely on self‐reports, which capture a first‐person perspective (Danese, 2020).

The use of prospective and retrospective measures in research and clinical practice has relied on the assumption that they identify the same individuals and can therefore be used interchangeably. However, a recent meta‐analysis has shown that this assumption is incorrect (Baldwin, Reuben, Newbury, & Danese, 2019). Over half of the individuals with prospective measures of maltreatment did not self‐report the maltreatment retrospectively and, similarly, over half of the individuals with retrospective self‐reports of childhood maltreatment did not have concordant prospective measures (Cohen's Kappa coefficient of agreement of 0.19) (Baldwin et al., 2019). This suggests that prospective and retrospective measures capture different underlying constructs.

The different measures of maltreatment also appear to be differentially associated with psychopathology. The well‐established link between child maltreatment and psychopathology is present among individuals with retrospective accounts of childhood maltreatment, regardless of whether they have consistent prospective measures (Danese & Widom, 2020; Newbury et al., 2018; Reuben et al., 2016; Shaffer, Huston, & Egeland, 2008). In contrast, the risk of psychopathology is minimal in individuals with prospective measures of childhood victimisation in the absence of retrospective self‐reports. This suggests that psychopathology is more strongly associated with the subjective appraisal that childhood maltreatment has occurred and/or with the ability to retrospectively recall these early experiences, rather than with exposure to the maltreatment per se (Baldwin, Coleman, Francis, & Danese, 2024; Danese & Widom, 2020, 2023).

Although the disagreement between prospective and retrospective measures of maltreatment and their differential associations with psychopathology have been recognised (Baldwin et al., 2019; Danese & Widom, 2020), the reasons for these differences have not been comprehensively mapped and are the focus of this review. We consider a range of mechanisms for low agreement, including measurement issues, memory processes, and motivational factors (summarised in Figure 1), before highlighting the implications of these mechanisms for developmental psychopathology and treatment research.

**Figure 1:**
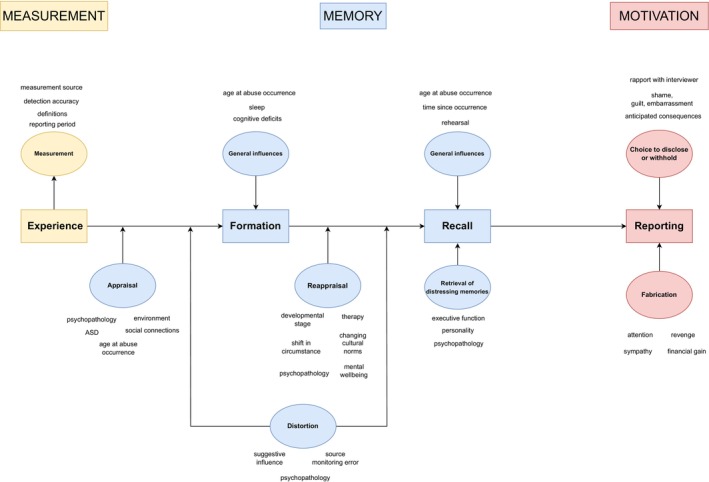
Explanatory mechanisms for disagreement in measures of childhood maltreatment

## Measurement

Poor agreement between retrospective and prospective measures may arise due to poor test–retest reliability and systematic differences between measures, such as definition, detection accuracy, source, and reporting period.

### Test–retest reliability

Retrospective measures of childhood maltreatment have imperfect test–retest reliability (Colman et al., 2016; Fergusson, Horwood, & Woodward, 2000; Langeland et al., 2015; Naicker, Norris, Mabaso, & Richter, 2017; Okeke, Wilkinson, & Roberts, 2017), although some studies reported high temporal stability in reporting (Nelson, Lynskey, Heath, Madden, & Martin, 2010; Yancura & Aldwin, 2009). For example, in a sample of 7,466 adults retrospectively reporting on adverse childhood experiences at two time points 12 years apart, 39% gave inconsistent reports (Colman et al., 2016). In an online survey on extra‐familial child sexual abuse conducted over a 4‐to‐6‐week period, 29.5% of respondents gave answers at Time 2 that were discrepant with their answers at Time 1 (Kappa coefficient of 0.39) (Langeland et al., 2015). These findings are important because low test–retest reliability adds error variance, ultimately reducing agreement between prospective and retrospective measures (Anastasi & Urbina, 1997).

### Definitions

Maltreatment definitions often vary for prospective and retrospective measures within the same study, therefore limiting comparability (Cicchetti & Manly, 2001). For example, child protection services (CPS) classification of maltreatment used to trigger social care and/or police actions relies on more stringent definitions than research criteria used to measure variation in caregiving experiences (Runyan et al., 2005). Furthermore, narrow prospective measures focusing on single types of abuse or neglect (e.g. sexual abuse, emotional neglect) do not capture the same constructs as broader retrospective measures of childhood maltreatment or adverse childhood experiences (Goodman, Quas, & Ogle, 2010).

### Detection accuracy

Prospective and retrospective measures have different strengths and limitations in their ability to detect maltreatment. Prospective measures based on official records likely have high sensitivity, namely the ability to detect true positive cases, because of the processes of substantiation used (Danese & Widom, 2020). However, official records likely also have a high false‐negative rate because only a small minority of children who have experienced maltreatment come to the attention of professionals (Baldwin et al., 2019; Sedlak & Broadhurst, 1996). In contrast, retrospective self‐reported measures can facilitate disclosure (Kim, Dubowitz, Hudson‐Martin, & Lane, 2008) and be valuable in identifying cases that are undetected by prospective measures (Kendall‐Tackett & Becker‐Blease, 2004). However, self‐reported measures (and particularly questionnaires) may, for example, also be more open to interpretation of the experiences investigated.

### Source

Prospective measures often rely on parent reports or official records, whereas retrospective measures are self‐reported. Such discrepancy in informants likely contributes to low agreement between maltreatment measures (Compier‐de Block et al., 2017; Goodman, De Los Reyes, & Bradshaw, 2010) in the same way it affects the agreement of other childhood psycho‐social measures (Nivison, Vandell, Booth‐LaForce, & Roisman, 2021; Sandberg et al., 1993; Stover, Hahn, Im, & Berkowitz, 2010; Tingskull et al., 2015). The discrepancy likely reflects limitations in professionals' and caregivers' awareness of young people's exposure to traumatic events (Ceballo, Dahl, Aretakis, & Ramirez, 2001; Stover et al., 2010) as well as individual appraisals of the experiences and motivations to report described subsequently.

### Reporting period

Prospective and retrospective measures might also capture different reporting periods leading to dilution of measure agreement. For example, a study by Denholm, Power, and Li (2013) compared prospective measures of childhood experiences up to 7 years of age with retrospective measures of experiences up to 16 years of age.

### Summary

Challenges with the measurement of maltreatment, such as imperfect test–retest reliability, varying definitions or detection accuracy, and differences in sources or reporting period between prospective and retrospective measures may contribute to measure disagreement. However, they are unlikely to fully explain the discrepancy because low agreement was also observed in studies with consistent measurement (Baldwin et al., 2019). Other possible explanatory factors involve memory and motivational processes.

## Memory

Retrospective reports of maltreatment might rely on second‐hand information that individuals receive from others (relatives, social care workers) and they incorporate into their life histories, even in the absence of autobiographical memories. Perhaps more often retrospective reports rely on individuals' autobiographical memories of events that occurred in childhood. Processes involved in the formation and recall of memories can lead to retrospective under‐ or over‐reporting and, ultimately, disagreement with prospective measures.

### Memory formation

Memory formation refers to the processes of encoding and consolidation that occur when a new memory trace is stored and integrated into pre‐existing representations (Paller & Wagner, 2002). Encoding generates the initial internal representation of an experience, whilst consolidation is the process by which representations are stored as memories and become more permanent and resistant to loss (Straube, 2012). Because encoding and consolidation are selective processes, not all experiences will be committed to long‐term memory or reflected in first‐person reports (Cowan, Schapiro, Dunsmoor, & Murty, 2021; Wilhelm et al., 2011).

Developmental factors, such as the age at which an event occurs, can affect memory encoding. Research indicates that adults' ability to recall memories of experiences occurring before 3 years of age is very limited (Pillemer & White, 1989). This phenomenon, known as infantile amnesia, may be due to the immaturity of the infant brain with insufficient functional competence to store memories (Campbell & Spear, 1972; Nelson, 2000), and/or an inability to subsequently recall them (Madsen & Kim, 2016; Rovee‐Collier & Cuevas, 2008; Travaglia, Bisaz, Sweet, Blitzer, & Alberini, 2016). Consistent with this, older age at the time maltreatment occurred might be associated with more detailed, complete, and reliable recollections of abuse (Greenhoot, McCloskey, & Glisky, 2005).

Memory consolidation is actively affected by sleep, as recently encoded memory traces are then reactivated and stabilised for long‐term storage (Rasch & Born, 2013). Sleep deprivation after training impairs memory performance for a range of tasks in humans and experimental animals (Smith, 1996; Smith & Rose, 1996). It is therefore possible that sleep problems, often observed in maltreated children (Javakhishvili & Widom, 2021; Schønning, Sivertsen, Hysing, Dovran, & Askeland, 2022), might compromise the consolidation of maltreatment memories and lead to under‐reporting in retrospective measures.

Memory formation can also be influenced by factors that affect the appraisal of events or distort the processes of encoding or consolidation.

#### Appraisal at formation

Memories are not objective accounts of events but interpretations coloured by subjective appraisal based on previous and current experiences (Bartlett, 1932). During formation, memories are labile and subject to interference (McKenzie & Eichenbaum, 2011), as either earlier memories can influence how new memories are encoded (proactive interference) and new information at the consolidation phase can change newly encoded memories (retroactive interference) (Straube, 2012). Individual differences in understanding, sensitivity to negative information, and propensity to interpret events as threatening or abusive might lead to contrasting interpretations of the same event during memory formation. Wider social and contextual factors can also help to shape individual responses to negative experiences.

Young children may not be able to conceptualise an experience as abusive (Stige, Halvorsen, & Solberg, 2020) or verbalise it (Halvorsen, Solberg, & Stige, 2020; Kolko, Brown, & Berliner, 2002; Schonbucher, Maier, Mohler‐Kuo, Schnyder, & Landolt, 2012), leading to impaired memory formation and underreporting in retrospective measures.

Individual differences in social cognition could influence the subjective appraisal of events and, therefore, reporting. For example, individuals with Autistic Spectrum Disorder (ASD) may subjectively experience a broader range of life events as traumatic in comparison to neurotypical individuals (Rumball, Happe, & Grey, 2020), and genetic liability for ASD in the population is associated with greater prevalence of self‐reports of childhood trauma (Peel et al., 2022; Ratanatharathorn et al., 2021; Warrier & Baron‐Cohen, 2021). Furthermore, individuals with high levels of neuroticism are more likely to appraise negative events as threatening and traumatic (Weinberg & Gil, 2016) and may, therefore, retrospectively self‐report more childhood adversities compared to prospective measures (Reuben et al., 2016). Psychopathology may also bias appraisal. For example, depression is associated with selective attention for negative personal experiences as well as biased interpretations of ambiguous personal situations in favour of negative inferences (Dalgleish & Werner‐Seidler, 2014). Similar biases in attention (Bar‐Haim, Lamy, Pergamin, Bakermans‐Kranenburg, & van Ijzendoorn, 2007; Dalgleish & Watts, 1990) and interpretation (Mathews, Richards, & Eysenck, 1989) have also been found for anxiety.

External factors may also contribute to how experiences of childhood maltreatment are appraised. The presence of supportive social relationships within and outside the family may reduce the likelihood that particular events are appraised as threatening or adverse in childhood (Smith & Pollak, 2021). Moreover, family or neighbourhood environments characterised by violence, chaos, or neglect may lead to normalisation of abusive experiences and, therefore, under‐reporting (Berger, Knutson, Mehm, & Perkins, 1988; Kruttschnitt & Dornfeld, 1992; Wekerle et al., 2001).

#### Distortion at formation

The ordinary links between experience and memory formation can also be disrupted in several ways so that memory formation may occur in the absence of experience. Poor encoding of information or compromised identification of the source of experience can lead to deficits in source monitoring, with the creation of false memories (McKenzie & Eichenbaum, 2011). One example of source monitoring error is the failure to differentiate between memories produced by external as opposed to internal events (i.e. reality monitoring), such as thoughts or dreams, because of similarities between how imagined and perceived experiences are encoded (Henkel & Franklin, 1998; Straube, 2012). Reality monitoring errors may lead to misattribution of thoughts or dreams about maltreatment to actual experiences, or vice versa (Schacter & Loftus, 2013). Source monitoring errors may also occur when an individual fails to differentiate between their own memories and external information (i.e. provided by others). Suggestive influences such as misleading, forced choice, or repeated questioning, can lead to the development of false beliefs or memories (Brewin & Andrews, 2017; Goodyear‐Smith, 2016; Loftus, 1996, 2005; Schacter, 1999). For example, in one study 70% of participants exposed to these interview techniques reported having committed a crime they did not commit (Shaw & Porter, 2015; Wade, Garry, & Pezdek, 2018). Concerns have also been raised about the practices of ‘memory recovery’ techniques during psychotherapy (Lindsay & Read, 1994). These types of source monitoring errors might lead to misattribution of informants' reports about maltreatment to an individual's own recollections, or vice versa.

Source monitoring errors like those reported above may be more common in children (Sutherland & Hayne, 2001) and adults with self‐reported lapses in memory and attention (Loftus, 2005) and particularly dissociative tendencies (Hyman & James Billings Jr., 1998; Sajjadi, Sellbom, Gross, & Hayne, 2021; Wright & Livingston‐Raper, 2002). Dissociation is described as disruption of the normal, subjective integration of behaviour, memory, identity, consciousness, emotion, perception, body representation, and motor control (American Psychiatric Association, 2013). Dissociation can be triggered by overwhelming emotions during exposure to traumatic experiences. It may take attentional resources away from the current experience with the creation of alternative experiences ranging from daydreaming to confusing and distressing discontinuity in the way people experience themselves and the world around them, and may disrupt the formation of traumatic memories (e.g. in dissociative amnesia) (Van der Kolk & Fisler, 1995). Dissociation has also been associated with fantasy proneness (Merckelbach, Otgaar, & Lynn, 2022), thereby potentially leading to distorted or false memory formation.

### Memory recall

Memory recall is the constructive process of accessing stored memories in response to external or internal cues. Factors that affect the accessibility of a memory for recall might also influence retrospective reports of childhood maltreatment. These factors include general predictors of memory recall over time and cognitive strategies people may use to avoid recall of upsetting memories. Furthermore, the way an experience is remembered over time may shift because of reprocessing of the stored memory through reappraisal and reconsolidation, and distortions during memory recall.

#### General influences on memory recall

The ability to recall memories of childhood maltreatment might reflect the domain‐general ability to recall any childhood memories (Clancy & McNally, 2005; Greenhoot et al., 2005; Henry, Moffitt, Caspi, Langley, & Silva, 1994), which diminishes over time because of the universal process of memory decay (Hardt, Nader, & Nadel, 2013) and increases with rehearsal.

Events occurring in the early years are more likely to be forgotten (Briere & Conte, 1993; Fergusson et al., 2000; Herman & Schatzow, 1987; Williams, 1994). Infantile amnesia may be partly attributable to underlying impairments in later memory recall (Callaghan, Li, & Richardson, 2014; Kim, McNally, & Richardson, 2006; Usher & Neisser, 1993). For example, a study in rats (Travaglia et al., 2016) suggested that early hippocampus‐dependent memories are not lost but stored as latent traces that can be recalled later when the right retrieval cue is presented. This forgetting may be adaptive as memory transience improves behavioural flexibility by removing unnecessary or outdated information (Norby, 2015; Richards & Frankland, 2017).

Another influence on memory recall is the degree of rehearsal (Bjork, 1988). Memory studies highlight the integral role of rehearsal and repetition in maintaining and strengthening memory over time (Campbell & Jaynes, 1966). Rehearsal can be influenced by several factors, including mood and personality. The mood congruency model states that negatively toned memories are more easily and frequently recalled when an individual is in a more negative mood (Blaney, 1986). This phenomenon has been found in experimental studies using mood induction procedures (Velten Jr., 1968) and in naturally occurring mood variations (Dalgleish & Werner‐Seidler, 2014). A meta‐analysis indicated that individuals with depression can recall up to 10% more negative word lists and stories than positive material in comparison to nondepressed individuals (Matt, Vazquez, & Campbell, 1992). In contrast, in nondepressed individuals, negative autobiographical events are generally more difficult to recall in comparison to positive events (Walker, Skowronski, & Thompson, 2003). Negative recall bias and faster access to negatively toned autobiographical information have also been associated with neuroticism (Bradley & Mogg, 1994; Costa & McCrae, 1980; Martin, Ward, & Clark, 1983), while extraversion has been related to the recall of more positive memories (Mayo, 1983; Schmidt, Jendryczko, Zurbriggen, & Nussbeck, 2023). Therefore, individuals with a history of low mood and neurotic personality traits may more frequently rehearse and more easily recall negative memories, potentially leading to over‐reporting of childhood maltreatment (Reuben et al., 2016).

#### Cognitive strategies influencing recall of distressing memories

Rehearsal is also central to the recall of distressing memories. On the one hand, memories of emotionally salient or distressing events can be remembered better than memories of neutral events (Kensinger & Schacter, 2008). This can be adaptive when the memory triggers defensive reactions in the face of life‐threatening dangers (LeDoux, 1998). On the other hand, distressing memories are undesirable negative experiences that can motivate avoidance strategies (Goodman, Quas, & Ogle, 2010). Dissociation, cognitive avoidance, and overgeneral memory are three such potential avoidance strategies.

Dissociation can not only be triggered during exposure to traumatic events (see above) but also by overwhelming emotions related to traumatic memories. This may, in turn, interfere with the rehearsal of traumatic memories and lead to inhibition of recollection (Ehlers & Clark, 2000).

Thought suppression is a common type of cognitive avoidance involving a deliberate attempt to avoid certain thoughts, which has been experimentally investigated using either the White Bear paradigm or the Think/No‐Think paradigm. Studies based on the White Bear paradigm showed that, when participants are asked to wilfully avoid thinking about a target thought (e.g. a white bear), they can access the avoided thought more easily – a phenomenon known as the post‐suppression rebound effect (Wegner, Schneider, Carter, & White, 1987; Wenzlaff & Wegner, 2000). This paradoxical effect of thought suppression is particularly evident in clinical samples (Purdon, 1999; Wegner & Zanakos, 1994) and has become a central tenet of cognitive models of psychiatric disorders, such as depression, anxiety, and Post‐Traumatic Stress Disorder (PTSD) (Abramowitz, Tolin, & Street, 2001). A more nuanced set of findings emerged when using the Think/No‐Think paradigm, in which participants are asked to repeatedly recall or suppress learned cue‐target pairings, which can be words, images, or even autobiographical material (Anderson & Green, 2001). Studies using the Think/No‐Think paradigm showed that certain individuals can successfully suppress retrieval of unwanted memories with no rebound effect, leading to forgetting over time (Anderson & Hulbert, 2021; Küpper, Benoit, Dalgleish, & Anderson, 2014; Noreen & MacLeod, 2014). Suppression‐induced forgetting is more common in individuals with good executive function (Levy & Anderson, 2008) and less common among those with depression, anxiety, and PTSD (Catarino, Küpper, Werner‐Seidler, Dalgleish, & Anderson, 2015; Dieler, Herrmann, & Fallgatter, 2014; Hertel & Gerstle, 2003; Stramaccia, Meyer, Rischer, Fawcett, & Benoit, 2021). Overall, the findings suggest that suppression‐induced forgetting may be a hallmark of mental well‐being and is reduced in individuals who have, or are at risk of developing, mental illness. Therefore, suppression may exert important influences on the retrieval of childhood maltreatment memories, possibly depending on individual differences in executive function and psychopathology risk.

Overgeneral memory can also contribute to cognitive avoidance by limiting recall of specific distressing memories in favour of more general memories, to buffer negative emotions (Ono, Devilly, & Shum, 2016; Sumner, 2012; Williams et al., 2007). Overgeneral memory is most often tested with the Autobiographical Memory Test (Williams & Broadbent, 1986), which requires participants to recall specific memories (i.e. if the event occurred once, at a specific time) after viewing cue cards with positive and negative words on them. Nonspecific recollection style (indicating over‐general memory) was reported in children (McCrory et al., 2017; Valentino, Toth, & Cicchetti, 2009) and adults (Dalgleish, Rolfe, Golden, Dunn, & Barnard, 2008) with histories of childhood maltreatment. Because the avoidance of specific negative experiences is repeatedly reinforced due to its affect‐regulating properties, rehearsal and recall of the memory can be reduced leading to under‐reporting of retrospective measures of childhood maltreatment.

#### Reappraisal at recall

Frederic Bartlett suggested that remembering does not emerge from passive recall of stored memories, but rather from an active, creative process of reconstruction, which pieces together fragments of past experiences guided by new experiences and views about ourselves and the world, or schemas (Bartlett, 1932). Bartlett's insight is supported by the more recent Multiple Trace Theory (MTT), which posits that memory traces enter a labile state during recall and require another consolidation period, or reconsolidation, before being committed again to long‐term storage (Nadel, Samsonovich, Ryan, & Moscovitch, 2000). During recall, the labile memory can be updated based on new information, current internal or external cues, and schemas (Alberini & LeDoux, 2013), potentially leading to different or new memories. The malleability of childhood maltreatment memories is supported by the poor intra‐rater reliability of self‐reports of childhood maltreatment over time described in some studies (Colman et al., 2016; Langeland et al., 2015). Reappraisals of childhood experiences in adulthood could lead to both underreporting and overreporting of childhood maltreatment, thereby increasing disagreement between retrospective and prospective measures.

Several internal and external factors may influence reappraisal at recall. First, more advanced cognitive and social development may enable adolescents and adults to reappraise childhood experiences with new interpretations, for example identifying abusive relationships that were previously normalised (Stige et al., 2020). Second, new experiences or learning can provide novel frameworks to interpret past experiences. For example, experiencing mental illness may promote the search for potential causes (causal search bias), thereby increasing reports of adverse childhood experiences by these individuals (Raphael & Cloitre, 1994; Susser & Widom, 2012). In contrast, therapeutic interventions (e.g. cognitive psychotherapy, such as cognitive restructuring or imagery rescripting) may promote a more positive reappraisal of adverse childhood experiences (Clark, 2013; Holmes, Arntz, & Smucker, 2007; Mancini & Mancini, 2018). Third, collateral information or suggestive comments (e.g. leading questions) from others, or changing cultural norms (e.g. about the boundaries of normative or abusive/neglectful relationships) may influence the reappraisal of recalled memories (Goodyear‐Smith, 2016; Lev‐Wiesel, Massrawa, & Binson, 2020).

In addition to these factors, the role of depression in memory reappraisal has been extensively examined. For example, depression at the time of recall was associated with a greater likelihood of reporting adverse childhood experiences that had not been previously reported as part of a longitudinal study (Colman et al., 2016). In a different study, currently depressed individuals described their parents in more negative terms compared to both a non‐depressed control group and remitted depressed individuals (Lewinsohn & Rosenbaum, 1987). Of note, remitted depressed individuals did not differ from never‐depressed controls in their recall of parental behaviour, suggesting that negative perceptions of the past were not a stable trait of individuals who had experienced depression but rather transient negative biases in appraisal (Lewinsohn & Rosenbaum, 1987). Furthermore, some longitudinal studies found small associations between the increase in depressive symptoms and greater reporting of childhood maltreatment over time (Goltermann et al., 2023; Yancura & Aldwin, 2009).

An alternative interpretation for the possible influence of depression on the recall of childhood experiences is that individuals with depression may provide more accurate accounts of their childhood experiences than non‐depressed individuals (Brewin, Andrews, & Gotlib, 1993), whilst non‐depressed individuals may provide positively biased and less accurate accounts (Coyne & Gotlib, 1983; Taylor & Brown, 1988). The Depressive Realism Hypothesis (Alloy & Abramson, 1979) and the Positive Illusion Theory (Taylor & Brown, 1988) depart from more traditional views by arguing that good mental health is associated with positively biased perceptions of experiences, with exaggerated self‐evaluations and perceptions of control and mastery, ability to reappraise negative experiences, and unrealistic optimism. Meta‐analytical evidence found only limited evidence for this phenomenon (Cohen's *d* = −.07) (Moore & Fresco, 2012).

#### Distortion at memory recall

During recall, memory may also be influenced by cognitive processes. For example, frequent rehearsal (e.g. re‐experiencing symptoms in PTSD) may progressively amplify traumatic memories by adding real or imaginary details to the original memory (Schacter, 1996). Such a ‘memory amplification’ phenomenon was observed in war veterans, refugees, and civilian research participants (Engelhard, van den Hout, & McNally, 2008; King et al., 2000; Mollica, Caridad, & Massagli, 2007; Oulton, Takarangi, & Strange, 2016; Roemer, Litz, Orsillo, Ehlich, & Friedman, 1998; Southwick, Morgan, Nicolaou, & Charney, 1997) so that greater PTSD symptom severity was associated with increased recall of trauma exposure over time. In contrast, 9/11 survivors who had consistently low or decreasing PTSD symptoms in the year after the attack remembered experiencing less subjective threat over time (Dekel & Bonanno, 2013). Similar cognitive processes may distort the recall of memories of childhood adversity.

### Summary

Retrospective reports of childhood maltreatment rely on experiences being encoded and consolidated into long‐term memory – general memory processes influenced by factors, such as age and sleep. The initial appraisal of an experience shapes its encoding, potentially leading to different memories of the same event. The link between experience and memory formation might also be disrupted or distorted, so that memory formation occurs in the absence of actual experience. Factors influencing memory accessibility for recall further complicate retrospective reports, including general predictors of recall and cognitive strategies to avoid distressing memories. Finally, memory is not static but can evolve over time due to the processes of reappraisal and reconsolidation, and is also susceptible to distortions during recall.

## Motivational factors

Even in the presence of accurate memories, maltreatment reporting can be influenced by motivational factors, leading individuals to under‐ or over‐report their experiences. Motivational factors encompass internal factors like shame or guilt, as well as interpersonal dynamics that affect reporting. These motivations may be intentional, with individuals taking deliberate actions affecting disclose, or unintentional, influencing reporting with no or limited individual awareness.

### Internal motivations

Internal factors like feelings of shame or guilt can act as barriers to disclosure. Abusive experiences during childhood can have a significant impact on a child's sense of self, view of the world, and interpersonal relations (Danese & Widom, 2021), and negative self‐conscious emotions can develop in the aftermath of maltreatment (Lewis & Wolan Sullivan, 2005; Spaccarelli, 1994). Self‐conscious emotions (e.g. empathy, shame, jealousy) relate to our self‐concept and awareness of how others might perceive and react towards us (Feiring, 2005). Two prominent negative self‐conscious emotions are shame and guilt, which tend to arise from a perceived violation of personal or social norms or standards of behaviour (Fletcher, 2011).

Individuals who have endured maltreatment tend to experience elevated and harmful levels of shame and guilt (Alessandri & Lewis, 1996; Fletcher, 2011). Shame is a state in which the self feels defective and damaged as a result of a perceived failure to meet a standard that is either socially or self‐imposed (Lewis, Alessandri, & Sullivan, 1992; Tangney, 1995). Guilt implies an individual has done something that they perceive to be wrong or deserved what happened to them: victims of abuse may feel guilty for aspects of what they might have done or failed to do before, during, or after an abusive experience (Fletcher, 2011). Shame and guilt may arise more often after childhood sexual abuse, particularly when there was no physical coercion in the sexual relationship (Noll, 2008; Paine & Hansen, 2002). In turn, negative self‐conscious emotions after abuse may contribute to delays in disclosure in maltreated children and adults, or concealment of the experiences (Lemaigre, Taylor, & Gittoes, 2017; McElvaney, Lateef, Collin‐Vézina, Alaggia, & Simpson, 2022; Morrison, Bruce, & Wilson, 2018; Taylor & Norma, 2013).

### Interpersonal dynamics

Interpersonal dynamics between the individual and potential recipient of a disclosure of maltreatment may influence the motivation to disclose (Jensen, Gulbrandsen, Mossige, Reichelt, & Tjersland, 2005). For example, trust is often compromised in individuals who have experienced child maltreatment (Neil et al., 2022) and may act as a barrier to disclosure in prospective or retrospective measures.

Detection of maltreatment by prospective measures partly relies on a child's decision to reveal abuse to caregivers or other significant adults (e.g. teachers, doctors, psychologists, social workers). This in turn relies on the quality and openness of a child's relationship with these adults (Bottoms, Goodman, Schwartz‐Kenney, & Thomas, 2002; Frijns, Finkenauer, Vermulst, & Engels, 2005; Jensen et al., 2005). Factors related to a better quality of parent–child relationships, such as youth‐rated trust in parents (Smetana, Metzger, Gettman, & Campione‐Barr, 2006), youth‐rated feelings of connectedness to parents, and parental warmth were associated with less secrecy and more disclosure from the child across a variety of domains (e.g. personal, peer, and schoolwork issues) (Darling, Cumsille, & Martinez, 2008; Tilton‐Weaver et al., 2010). In contrast, threats might inhibit disclosure and detection in prospective measures. Direct threats of violence or other negative consequences to the victim or their families can be effective means of silencing children (Berliner & Conte, 1990; Paine & Hansen, 2002; Schaeffer, Leventhal, & Asnes, 2011). Indirect threats, such as the risk of the child being taken away from their parents (if the abuse is intra‐familial), family disintegration, divorce, or perpetrator imprisonment may also reduce disclosure by children or their parents (Bottoms et al., 2002).

Interpersonal factors may also play a role in facilitating or inhibiting retrospective disclosure of personal experiences of maltreatment to an interviewer. Lack of rapport or trust in the interviewer may lead to under‐reporting of maltreatment in adulthood compared to previous records or interview data (Della Femina, Yeager, & Lewis, 1990). An individual may be embarrassed or unwilling to disclose maltreatment if they have insufficient rapport with the interviewer (Widom & Morris, 1997). Men are less likely to retrospectively report documented childhood sexual abuse than women (Widom & Morris, 1997), which might reflect embarrassment or gendered differences in perceptions of experiences. Reports may also be influenced by social desirability bias (i.e. the tendency to answer questions in a way that others will view favourably) (Fisher, Bunn, Jacobs, Moran, & Bifulco, 2011; Straus, Hamby, Finkelhor, Moore, & Runyan, 1998), as well as the level of privacy within which an interview or questionnaire is completed (Langeland et al., 2015). The anticipated negative consequences may be less likely to play a role in retrospective measures, because of the lack of mandatory reporting to social services for adult self‐reports as opposed to disclosures in childhood (Tajima, Herrenkohl, Huang, & Whitney, 2004). However, the anticipated consequences of reporting maltreatment might be exploited by individuals deliberately fabricating abuse, motivated by some form of personal gain, such as seeking retribution or revenge (e.g. against a parent, family member, ex‐boyfriend, spouse, etc) (Goodyear‐Smith, 2016); obtaining sympathy or attention (Engle & O'Donohue, 2012); or financial gain through victimhood (Goodyear‐Smith, 2016). Of note, however, national studies estimate that around only 4% of cases of child maltreatment that come to the attention of the authorities are considered to be intentionally fabricated (Oates et al., 2000).

### Summary

The accuracy of maltreatment reporting, whether through prospective or retrospective measures, is contingent on the individual's motivation to disclose, withhold, or fabricate information. Various barriers inhibit disclosure, including internal factors, such as feelings of shame or guilt, as well as broader contextual factors like interpersonal dynamics between the individual and the recipient of the disclosure.

## Implications

We aimed to provide a comprehensive map of key potential mechanisms underlying the disagreement between prospective and retrospective measures of maltreatment. Empirical research is needed to directly test the hypotheses presented here and to identify additional mechanisms that we might have omitted. A better understanding of the mechanisms underlying differences between prospective and retrospective measures of maltreatment could provide novel insights into the underlying constructs, measurement of maltreatment, mechanisms of resilience and vulnerability to psychopathology, and treatment strategies.

### Constructs

The disagreement found between prospective and retrospective measures of childhood maltreatment suggests that the two measures do not capture the same underlying construct. Prospective measures typically capture a third‐person perspective from informants. As such, they can be influenced by several factors including informants' knowledge of the event, their definition of maltreatment, and their motivation to disclose. Some prospective measures (e.g. official court records) can be perceived as more objective, in that they summarise the opinions of several different professionals who have undertaken in‐depth investigations, and are used as the legal standards to protect children and prosecute perpetrators. However, they are not perfect measures because they do not capture all cases of maltreatment in the population (Danese & Widom, 2020).

Retrospective measures, in contrast, capture a first‐person, subjective perspective from individuals. As displayed in Figure 1 and discussed above, they can be influenced by the individual's appraisal of the event, their memory processes (formation, distortion, reappraisal, decay), and their motivations to disclose the events. Because of their subjective nature, retrospective measures have been denigrated and diminished in the past as poor indicators of whether maltreatment occurred. However, they capture the lived experience of individuals and provide key insights into their beliefs and schemas.

### Measurement

The information captured by current retrospective measures of childhood maltreatment is typically limited to the basic disclosure of relevant childhood experiences. However, the subjective experience of individuals is likely much richer and more nuanced, and a better understanding of it could provide new insights to improve measurement. A more detailed phenomenological description of the subjective experience of childhood maltreatment could be developed based on the cognitive psychology framework for traumatic memories in the context of PTSD. For example, it may be helpful to consider the organisation (Ehlers & Clark, 2000), intrusiveness (Michael, Ehlers, Halligan, & Clark, 2005), centrality (Berntsen & Rubin, 2006), and stability (Dekel & Bonanno, 2013) of the memories, and the nature of trauma‐related cognitions (Foa, Ehlers, Clark, Tolin, & Orsillo, 1999; Meiser‐Stedman, Smith, Yule, & Dalgleish, 2007).

To access information about the subjective experience of childhood maltreatment, researchers and clinicians will also need to acknowledge and address self‐conscious emotions and interpersonal dynamics in the context of the assessment. Because of the intimate nature of these experiences, it will be important to co‐develop new instruments and assessment protocols with survivors of childhood maltreatment. The aim of these innovations in retrospective measures of childhood maltreatment is not to increase the agreement with prospective measures but to recognise and comprehensively map the subjective experience of survivors.

### Resilience and vulnerability

This literature review found that similar characteristics may affect both the agreement between prospective and retrospective measures of childhood maltreatment and the risk for trauma‐related psychopathology. For example, personality traits of high neuroticism and low extraversion may negatively bias the interpretation of experiences with formation of more negative memories (Weinberg & Gil, 2016) and favour recall and rehearsal of negative versus positive memories (Costa & McCrae, 1980; Martin et al., 1983; Mayo, 1983). Autistic Spectrum Disorder may predispose individuals to interpret a broader range of life experiences as traumatic (Rumball et al., 2020). In contrast, good executive function may promote forgetting because of heightened cognitive flexibility and cognitive inhibition (suppression) of memories (Levy & Anderson, 2008). Supportive social relationships may also reduce the likelihood of perceiving an event as threatening (Smith & Pollak, 2021). Intriguingly, the same characteristics have been previously identified as vulnerability or resilience factors for trauma‐related psychopathology (Collishaw et al., 2007; Kerns, Newschaffer, & Berkowitz, 2015; Mary et al., 2020; Polak, Witteveen, Reitsma, & Olff, 2012; Sayed, Iacoviello, & Charney, 2015). Consistent with popular cognitive models of emotional disorders (Beck, 2002), increased access to negative memories may influence the onset and maintenance of psychopathology. As such, a better understanding of factors influencing the agreement between prospective and retrospective measures may provide new insights into the mechanisms underlying stronger associations of the subjective vs objective experience of childhood maltreatment and psychopathology (Baldwin et al., 2024; Danese & Widom, 2020, 2023). Future research will be necessary to directly test the factors discussed above as potential mediators of the associations between the subjective experience of childhood maltreatment and psychopathology. To enable this work, future research should capitalise on longitudinal datasets with repeated measures of psychopathology collected before and after the assessment of the subjective experience, to disentangle the contributing role of recall bias and the continuity in psychopathology in any observed associations (Danese & Widom, 2023).

### Treatment

If retrospective recall of childhood maltreatment is a risk factor for psychopathology, then memory therapeutics targeting relevant memories and broader features of the subjective experience may prove beneficial to prevent or treat maltreatment‐related psychopathology. Current evidence‐based treatments for PTSD, such as Trauma‐Focused Cognitive‐Behavioural Therapy (Cohen & Mannarino, 2008), already address avoidance of trauma‐related memories reducing their recurrence and challenge memory distortions and unhelpful trauma‐related cognitions (e.g. shame, guilt). Such interventions, however, are only used when PTSD is a primary concern and could be tested in the treatment of other psychiatric disorders.

Novel memory therapeutics (Dalgleish & Hitchcock, 2023) might help reduce the risk of maltreatment‐related psychopathology in four ways. First, traumatic memories can be restructured through imagery rescripting or visuo‐spatial cognitive distraction (e.g. Tetris). Second, to reduce harmful affective biases in memory recollection (i.e. negative memory biases and diminished access to positive memories), selective emphasis can be placed on recollecting and savouring positive memories. Third, to reduce overgeneral biases in memory recall, individuals can be trained to deliberately and intentionally recall specific memories in response to word or picture cues. Finally, work in animal models suggests that it might one day be possible to target memory consolidation or reconsolidation through pharmacological agents or behavioural strategies (Frankland, Josselyn, & Köhler, 2019; Monfils & Holmes, 2018; Phelps & Hofmann, 2019).

## Conclusions

Childhood maltreatment contributes to a large mental health burden worldwide. Different measures of childhood maltreatment are not equivalent and capture meaningful differences in maltreatment experiences and their impact on psychopathology. Beyond the important methodological concerns, differences between prospective and retrospective measures of childhood maltreatment likely reflect cognitive and motivational processes. A better understanding of these processes might increase our ability to mitigate risk of maltreatment‐related psychopathology.

**Key points**

- Prospective and retrospective measures of maltreatment identify different groups of individuals, and those with retrospective measures have greater risk for psychopathology.
- This review presents a comprehensive framework of factors contributing to such measurement disagreement, including methodological issues, memory influence, and disclosure motivation.
- Greater understanding of measurement disagreement informs our understanding of the constructs and measurement of maltreatment.
- Characteristics influencing both measurement disagreement and risk of trauma‐related psychopathology suggest potential underlying mechanisms and targets for interventions to mitigate trauma‐related psychopathology.

## Data Availability

Data sharing not applicable – no new data generated, or the article describes entirely theoretical research.
